# Atlantic mackerel ( *Scomber scombrus* ) change skin colour in response to crowding stress

**DOI:** 10.1111/jfb.14987

**Published:** 2022-01-23

**Authors:** Guro M. Tveit, Neil Anders, Morten S. Bondø, John R. Mathiassen, Mike Breen

**Affiliations:** ^1^ Department of Seafood Technology SINTEF Ocean AS Trondheim Norway; ^2^ Fish Capture Division Institute of Marine Research (IMR) Bergen Norway

**Keywords:** Atlantic mackerel, crowding, image analysis, iridophore, skin colour, stress

## Abstract

Wild capture can be stressful for fish. Stress has the potential to induce mortality in released unwanted catches or negative flesh quality consequences in retained ones. Such effects compromise sustainable natural resource management and industry profitability. Mitigating stress during capture is therefore desirable. Biological indicators of stress can objectively inform fishers as to the functional welfare status of catches during fishing operations. If they are to be of practical use in mitigating stress during wild capture events, such indicators must be quantifiable, respond rapidly, reflect the level of induced stress and be easily observable. Atlantic mackerel (*Scomber scombrus*) are extensively targeted by purse seine fisheries in European waters but are particularly vulnerable to stress. Excessive crowding in the net is thought to be the principal stress mechanism. There is therefore a need to develop indicators of crowding stress for this species so that catch welfare can be improved. Here, we demonstrate that *S. scombrus* exhibit a skin colour change from predominately green to predominately blue when exposed to crowding stress. In sea cage trials, we induced various degrees of stress in groups of wild‐caught *S. scombrus* by manipulating crowding density and its duration. Skin colour was quantified in air using digital photography. The colour change occurred rapidly (within the typical duration of crowding events in the fishery), and its magnitude was correlated to the severity and duration of crowding. Bluer fish were also associated with higher levels of plasma lactate. No appreciable colour change was observed in uncrowded (control) groups during the treatment period. Nonetheless, unstressed *S. scombrus* did turn blue <1 h after death. Together, these results indicate that skin colour change has the potential to be a useful real‐time indicator of crowding stress for *S. scombrus* and could therefore be used to improve welfare during wild capture fishing.

## 1 INTRODUCTION

Wild capture fishing can be stressful for the captured fish. Stressors are typically acute (relatively short term) and may include rapid changes in ambient conditions (*e.g*., temperature and depth), physiological disturbance, exhaustion and/or different types and severity of injuries (Breen *et al*., 2020). Such stressors can result in undesirable outcomes. The release of unwanted catch is a routine component of wild capture fishing (Zeller *et al*., 2018). Nonetheless, stressors act to reduce the survival potential of released catches. This is of concern for marine resource management as any resulting mortality may be unaccounted for in stock assessment (Gilman *et al*., 2013), as well as being ethically unjustifiable. For retained catches, stressors can result in undesirable flesh quality consequences (Poli *et al*., 2005) that may reduce the value of catches (Sogn‐Grundvåg *et al*., 2021 and references therein). A reduction in stress during capture may therefore promote not only individual fish welfare but also lead to greater sustainability in marine resource management and increased profitability for the industry. We refer to welfare in functional terms, meaning that welfare is good when the animal is able to cope with challenges from its environment within its biological capacity (Diggles *et al*., 2011).

Due to the animal's inability to relate any subjective experience, indicators of stress are required if negative capture‐related welfare impacts are to be reduced. To be informative, such indicators should: (a) be quantifiable (to enable objective inference); (b) correlate to the amount of stress received (also allowing objective inference); (c) respond rapidly to stress (to allow timely mitigation action that benefits the welfare of the catch); and (d) be practical to measure in real time during fishing operations that are typically dynamic (*see* Noble *et al*., 2018).

Although relatively benign in comparison to other capture methods (Veldhuizen *et al*., 2018), fish captured by purse seine can still experience considerable negative welfare implications. After a school of fish is surrounded by the gear, the capture process involves a progressive increase in the crowding density of the fish, as net volume is reduced to concentrate the catch and facilitate its transfer aboard the vessel (Ben‐Yami, 1994). Of the species targeted by the extensive and commercially important purse seine fisheries of Europe (ICES, 2020), Atlantic mackerel (*Scomber scombrus* L.) are particularly vulnerable to crowding stress. For instance, crowding of this species has been associated with dermal injury (Anders *et al*., 2021; Lockwood *et al*., 1983), physiological disturbance (Anders *et al*., 2020, 2021; Lockwood *et al*., 1983; Swift, 1983), behavioural impairment (Anders, Breen, *et al*., 2019; Anders, Howarth, *et al*., 2019; Handegard *et al*., 2017), reductions in flesh quality (Anders *et al*., 2020; Digre *et al*., 2016) and excessively high rates of delayed mortality in released catches (Huse & Vold, 2010).

Existing EU and Norwegian purse seining legislation attempts to minimise crowding stress by enforcing a limit after which *S. scombrus* cannot be released (defined by a proportion of net length hauled onboard, Marçalo *et al*., 2019). However, variable net lengths between vessels and variable catch sizes between casts mean that densities may exceed safe levels even when fish are released in a regulation‐compliant manner (Tenningen *et al*., 2019). Furthermore, schools of fish are unlikely to adopt densities solely dependent on net volume (Handegard *et al*., 2017), and localised areas of high density (due to deformations in net shape) can arise relatively early in the capture process (our unpublished observations). There is therefore a need to develop crowding stress indicators for *S. scombrus* that better reflect the actual capture conditions experienced by the fish.

The dorsal surface of *S. scombrus* consists of alternating dark and light stripes which extend laterally onto the upper flanks (Denton & Rowe, 1998). The light stripes are typically green/yellow in colour but can change to blue when the animal is exposed to stressors, including crowding (Holeton *et al*., 1982; Lockwood *et al*., 1983; Pawson & Lockwood, 1980; Swift, 1982, 1983). As this change is reported to be readily apparent and to occur soon after stressor onset, skin colouration has potential as a stress indicator for use during purse seining. However, to date, this colour change has only been reported in the literature incidentally and in a qualitative manner. Furthermore, these earlier studies utilised fish that were highly likely to be stressed due to capture and handling procedures. Consequently, it is unknown whether the colour change occurs in response to crowding stress alone, whether it corresponds to the degree of stress received, how soon it occurs and whether it can be objectively quantified and interpreted. These criteria are key if skin colour change is to have utility as a stress indicator.

In this study, we examine skin colour change in*S. scombrus* in response to crowding stress. Wild‐caught groups of *S. scombrus* were exposed to various durations and severities of crowding in sea cage trials, with skin colour being assessed quantitatively using image analysis. The work was conducted as part of a larger experiment in which a benign husbandry technique was used to minimise captivity/handling stress, thereby allowing the examination of various physiological stress responses and post‐crowding survival rates (reported in Anders *et al*., 2021). The intention of the current study was to determine the usefulness of skin colour change as an indicator of crowding stress. Establishing skin colour as a stress indicator may facilitate informed decisions by fishers during purse seine fishing operations so that negative welfare outcomes such as mortality (in released catches) and reductions in flesh quality (in retained catches) can be minimised. Specifically, the authors aimed to address the following research questions: (a) Do *S. scombrus* exhibit a skin colour change in response to crowding stress?; (b) Does the degree of any colour change correlate to the amount of stress received? and; (c) Does any colour change occur within the typical time frame of crowding events in the fishery?

## 2 MATERIALS AND METHODS

### 2.1 Fish capture and husbandry

During the summer of 2018 and 2019, wild adult *S. scombrus* were benignly attracted into a large (12 × 12 × 12 m) aquaculture net cage (anchored *c*. 100 m offshore) at the Austevoll Research Station, Norway (60°N) using feed pellets. Fish were held there for *c*. 10 months (May/June trials) or *c*. 1.5 months (August/September trials) prior to beginning trials. Fish fed *ad libitum* on natural prey items from the cage, supplemented with feed pellets as required.

The crowding trials took place in one of two experimental cages (5 × 5 × 5 m with a pyramidal bottom; total volume 149.17 m^3^) situated inside a larger net pen. For this, groups of approximately 100 fish were benignly transferred into each of the experimental cages, again by encouraging them to enter using feed pellets. Groups were given a minimum of 48 h to acclimatise to experimental pens before beginning the trials. Full details of capture and husbandry methods are described in Anders *et al*. (2021).

### 2.2 Ethical statement

All husbandry and experimental protocols were pre‐authorised by the Norwegian animal welfare authority (Mattilsynet, FOTS licence ID: 19238).

### 2.3 Crowding trials

A total of eight crowding trials were conducted. The intensity and duration of exposure to a stressor largely determines the resulting allostatic load (Korte *et al*., 2007). We therefore applied various crowding densities and durations (Table 1) to manipulate the amount of crowding stress received by fish. The applied densities were intended to simulate different possible densities that fish may experience in the purse seine fishery, ranging from previously established sub‐lethal levels (Handegard *et al*., 2017) through to the densities expected for large catches at the end of the capture process (Tenningen *et al*., 2019). The applied durations were intended to simulate typical fishery crowding durations for either released catches (*c*. 15 min, Anders, Breen, *et al*., 2019) or retained catches (*c*. 60 min, during which catches are pumped onboard; Digre *et al*., 2016).

**TABLE 1:** Pertinent details of trials conducted to assess the response of Atlantic mackerel (*Scomber scombrus*) to crowding stress. Groups of wild‐caught *S. scombrus* were crowded at different densities and durations in sea cages

Period	Trial name	Dates	Treatment duration (decimal hours)	No. fish exposed to treatment	No. of fish sampled for colour during treatment	Mean (± 95% c.i.) fork length^a^ (cm)	Mean weight (± 95% c.i.)^a^ (g)	Pre‐treatment density (kg/m^3^)	Mean (± 95% c.i.) pre‐treatment oxygen concentration (mg/L)	During‐treatment density (kg/m^3^)	Mortality proportion (95% c.i. ^b^)
May/June	Control 1	21 May 2019–21 May 2019	1.87	149	16	38.57 ± 0.97	816.57 ± 54.51	0.76	10.10 ± 0.01	0.76	No survival monitoring
	Control 2	28 May 2019–6 June 2019	0.68	116	10	39.33 ± 1.09	830.20 ± 77.16	0.61	9.89 ± 0.002^c^	0.59	0.00 (0.00, 0.04)
	High & Prolonged 1	22 May 2019–22 May 2019	1.13	78	22	38.13 ± 1.12	752.43 ± 67.53	0.41	10.95 ± 0.003^c^	(182.75^d^)	No survival monitoring
	Low	29 May 2019–6 June 2019	0.25	231	8	37.77 ± 1.30	719.23 ± 52.19	1.19	9.88 ± 0.002	92.00	0.00 (0.00, 0.02)
	High & Prolonged 2	6 June 2019–6 June 2019	1.15	91	NA^e^	37.95 ± 0.83	730.20 ± 66.68	0.45	9.62 ± 0.001	182.75	No survival monitoring
August/September	Control 3	21 August 2019–17 September 2019	0.75	175	10	38.40 ± 0.83	677.47 ± 41.34	0.78	8.36 ± 0.002	0.76	0.00 (0.00, 0.02)
	Moderate	22 August 2019–9 November 2019	0.22	131	7	38.17 ± 1.18	646.00 ± 71.05	0.59	7.86 ± 0.01	146.21	0.03 (0.01, 0.07)
	High	28 August 2019–17 September 2019	0.25	150	9	38.64 ± 0.93	678.79 ± 62.10	0.64	8.24 ± 0.007	179.87	0.31 (0.23, 0.39)

^a^ Calculated from fish sampled prior to or during treatment only.

^b^ Confidence intervals calculated using Wilson score intervals.

^c^ Measured <1.5 h after treatment period.

^d^ Data not collected due to logistical constraints on the day of the trial. Therefore, density assumed to be the same as for the “High & Prolonged 2” trial.

^e^ Skin colour data not collected due to logistical constraints on the day of the trial but reported here because of the supporting data it provides regarding crowding density.

Crowding was achieved by lifting experimental cages vertically in the water by hand. This restricted the available swimming volume and concentrated fish into the pyramidal section at the bottom of the cage. The procedure for reaching the desired density took *c*. 5 min. Fish were then kept in a crowded condition for the desired amount of time before the cages were released, allowing them to return to the maximum volume by sinking under their own weight. Cage volume was not changed for control trials. Mortality rates (Table 1) were assessed for up to 27 days post‐crowding. Full details of survival monitoring are reported in Anders *et al*. (2021). Note that for the “high & prolonged” trials and their control (“Control 1”, Table 1), we terminated the experiment (without monitoring survival) by euthanising all fish immediately following the crowding period. This was done for fish welfare reasons and as required by the national animal welfare authority (Mattilsynet). In total, the authors successfully monitored fish for skin colour change in three control trials, in one “high & prolonged” crowding trial and in one trial each at low, moderate and high crowding densities (Table 1). Note that a second “high & prolonged” crowding trial was conducted, but no skin colour data were collected due to logistical constraints.

A lack of suitable instrumentation meant we were not able to obtain real‐time estimates of crowding density. We therefore estimated density *post‐hoc* from a video camera situated *c*. 1.6 m above the water line. Assuming the schools adopted a density dependent on the available cage volume, we quantified *et al*., 2012). Under the assumption that the pyramidal section formed a right cone shape, volume was then calculated from this area value and the trigonometrically derived water depth [refer to Anders *et al*. (2021) for further details]. Due to technical challenges, video was not collected during the “High & Prolonged 1” trial (Table 1). We therefore assumed that crowding density during this trial was the same as for the “High & Prolonged 2” trial. Based on the qualitative observation of the fish density during crowding, this assumption was deemed acceptable for the purposes of this study.

### 2.4 Collection and sampling of fish

Dependant on the trial, we collected fish from cages prior to crowding (up to five fish), during crowding (as many as possible within the treatment period) and after crowding (up to five fish at each of the following time points: 2 h post‐crowding, 24 h post‐crowding and at trial termination, *i.e*., the end of the survival monitoring period). No pre‐crowding samples were collected for the “High & Prolonged” trials, and no post‐crowding samples were collected for trials in which survival was not monitored (Table 1). Fish collected during crowding periods (as well as any moribund individuals which were therefore easy to catch) were collected using a landing net. Outside of this, the large, unrestricted volume of the cages meant that collection by landing net was not possible. In these scenarios, we therefore encouraged feeding behaviour with pellets and collected fish using a barbless hook (size: #1/0) and handline. Hooked fish were immediately removed from the cage, and any additional stress induced by collecting fish in this manner is likely to be minor in comparison to the crowding‐induced effects. This way of collecting samples was therefore deemed acceptable in the absence of any other viable collection technique. Full details of sample collection are contained in Anders *et al*. (2021).

Collected fish were sampled sequentially as follows: (a) assessed for behavioural vitality (with alternate fish also being assessed for in‐water vitality first, results not reported here); (b) euthanised using a percussive blow to the head; (c) both dorsal sides photographed for skin colour assessment (see details below); (d) total weight recorded; (e) blood collected from the caudal vasculature and muscle pH recorded (results reported in Anders *et al*., 2021); and (f) fork length recorded to the nearest centimetre below. Vitality assessment involved rapidly assessing the animal for the presence or absence of a suite of behavioural/reflex indicators. All effort was made to make sampling as quick as possible; the whole procedure typically took *c*. 1 min.

The collected blood was immediately chilled on ice, and later processed to extract plasma which was stored prior to laboratory analysis at −80°C. The details of blood collection and analytical protocols are reported in Anders *et al*. (2021). In short, the blood analysis revealed that *S. scombrus* exposed to crowding exhibited a typical teleost acute physiological stress response characterised by elevated levels of cortisol, glucose and lactate (Anders *et al*., 2021). Hereafter in this work, we examine blood physiology (*i.e*., cortisol, lactate and glucose) in terms of its correlation to individual *S. scombrus* skin colour.

### 2.5 Photography and colour analysis

Due to logistical constraints, skin colour change was only assessed in seven of the eight trials (Table 1). The camera used for photographing skin was a 36.3‐megapixel FX‐format Nikon D800E, with a Nikon 35 mm f/1.8 G FX AF‐S lens and a Nikon Speedlight SB900 full‐spectrum flash. Both the lens and flash had mounted linear polarising filters, crossed at 90 degrees relative to each other. The purpose of the polarising filters was to remove specular reflections and to provide more accurate colour imaging of the skin. Images were acquired with full manual settings on the camera (1/250 s exposure, ISO 100, F/5.6) and exported in NEF RAW format for post‐processing.

An X‐rite ColourChecker was photographed by the same equipment/settings, and a custom colour correction profile was created and applied to the RAW‐photos during post‐processing using Adobe Lightroom software. Exposure variations due to Nikon speedlight flash inconsistencies between images were corrected on each image, by measuring the intensity of the white background the fish was photographed on. Images were then cropped, resampled and exported in JPEG format for final processing and analysis in LabVIEW software (NI, Austin, Texas, USA).

**FIGURE 1:**
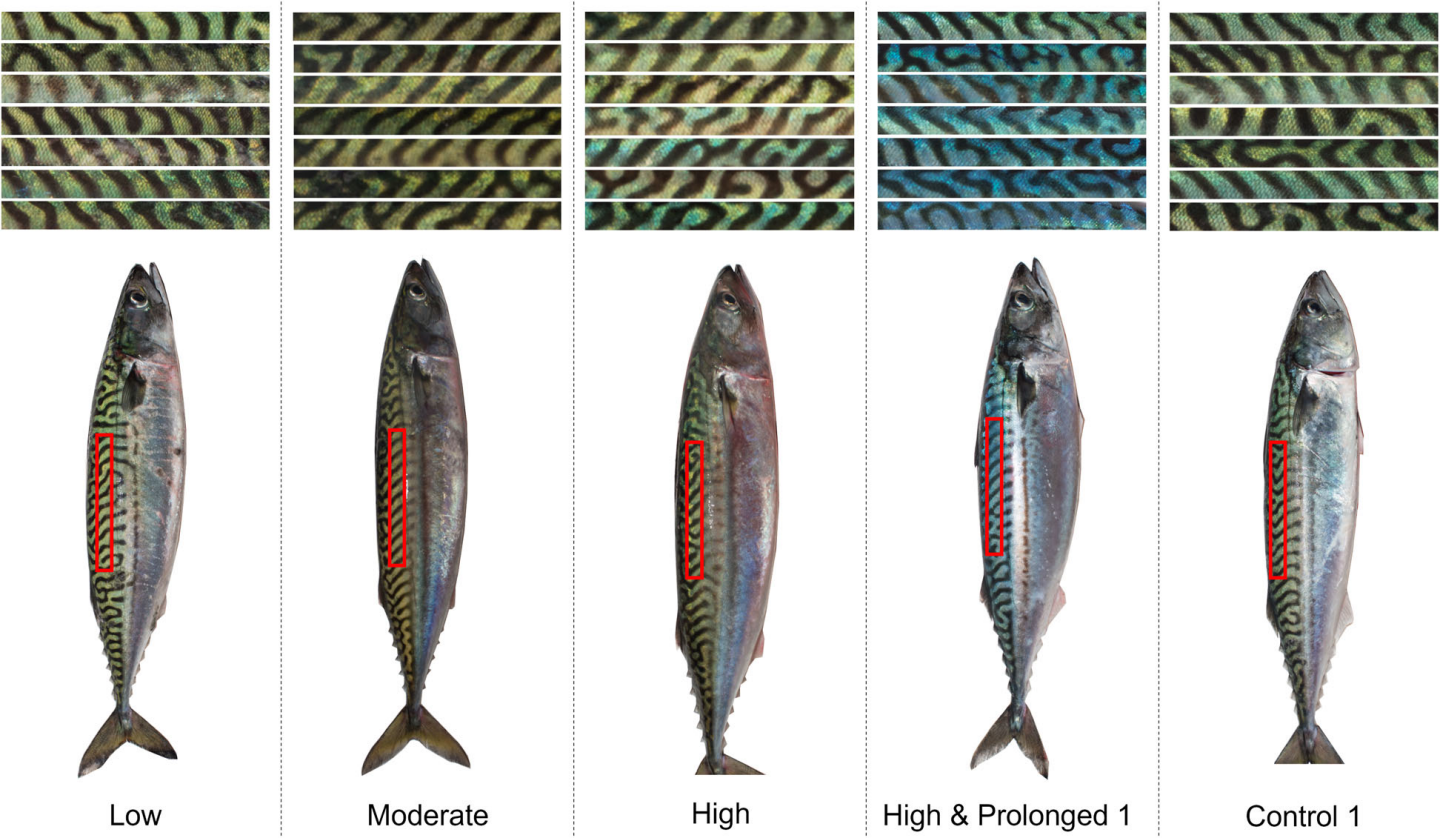
The response of Atlantic mackerel (*Scomber scombrus*) dorsal skin colour to various degrees of simulated crowding stress in sea cages. The upper panel shows mosaics of example dorsal areas, with crowding exposure time increasing from top to bottom. The lower panel shows photographs of one fish from each treatment, with the approximate colour analysed thumbnail region annotated in red. Individuals were photographed in air throughout stressor exposure under standard lighting conditions

For the final image analysis, two LabVIEW programmes were developed. The first programme was a user interface where a rectangular 530 × 53 pixels area on the right, dorsal side of the fish was manually selected and exported as an RGB‐thumbnail (as shown in the bottom of Figure 1). The second programme converted these thumbnails into CIE L*a*b* colour space values. CIE L*a*b* colour space was considered appropriate because it approximates human vision with an axis (b*) that can be used to quantify the expected blue colour change (Luo, 2016). A threshold was applied from the green colour plane of the RGB‐thumbnail so that only the pixels of the coloured section of the fish were selected, excluding the dark stripes. The mean L* (colour lightness), a* (green‐red component) and b* (blue‐yellow component) of the subset of pixels was then calculated. The image analysis was conducted “blind”; that is, the analyst had no prior knowledge of the status of the fish or its treatment group (with the exception of the post‐mortem skin colour trial).

### 2.6 *Post‐mortem* skin colour

*Scomber scombrus* skin colouration is reported to turn blue after death, irrespective of pre‐mortem condition (Swift, 1983). Dependant on how rapidly this occurs, such an effect has the potential to limit the usefulness of skin colour change as an indicator of any stress that occurred in the *pre‐mortem* phase. We therefore collected an additional 15 (uncrowded) fish from the large net pen using the handline method to assess how quickly after death *S. scombrus* turn blue. These fish were immediately euthanised with a percussive blow to the head followed by brain spiking. Fish were then stored in water and repeatedly photographed, as previously described, at the following time points *post‐mortem*: 0, 1, 4 and 24 h. To control for effects of exposure to sunlight (in particular UV radiation) on the skin pigments, subsets of these fish were stored in either dark or light conditions.

### 2.7 Statistical analysis

All statistical analysis was conducted in R (version 3.6.2), with a significance level of *P* < 0.05. For each colour component (L*, a* and b*), we fitted separate generalised least squares (GLS, Zuur *et al*., 2009) models to investigate whether crowding influenced skin colour during and after stressor exposure. We considered the following predictor terms as main effects: (a) “trial” (categorical: “Control 1,” “Control 2,” “Control 3,” “High & Prolonged 1,” “Low,” “Moderate” or “High”); (b) “monitoring period” (categorical: “Pre‐treatment,” “Treatment,” “2 h post‐treatment,” “24 h post‐treatment” or “Termination”); and (c) “vitality” (categorical: “vitality assessed” or “vitality not assessed”). The “vitality” term was dropped from models if not significant.

To determine whether the degree of any blue‐yellow (b*) skin colour change correlated to the amount of stress received, we fitted another GLS model to data collected during exposure to crowding only. This model considered an interactive effect between: (a) “trial” (categorical: as detailed above) and (b) “exposure” (continuous: time since treatment start). Temporal autocorrelation structures did not improve model fit and were therefore not included (Zuur *et al*., 2009).

To investigate whether skin colour change correlated to physiological stress, we employed generalised additive mixed‐effects models (GAMM). Additive models allow for non‐linear relationships between predictors and response (Zuur *et al*., 2009). Separate models were fitted to describe the relationship between the b* colour component and (a) plasma lactate; (b) glucose; and (c) cortisol, with Gaussian (link function = identity) error structures, cubic regression splines and the basis dimension selected by AIC (Zuur *et al*., 2009). We modelled “monitoring period” nested within “trial” as random intercept effects to account for non‐independence of b* values sampled within these (hierarchical) factors and to allow population‐level effects to be determined (Zuur *et al*., 2009).

The stability in *post‐mortem* skin colour (in terms of the b* colour component) was investigated using a linear mixed‐effect (LME) model. For this, we fitted main effects of: (a) “time *post‐mortem*” (categorical: “0 h,” “1 h,” “4 h” or “24 h”) and (b) “storage” (categorical: “light” or “dark”). The “storage” term was dropped if not significant. Individual‐level random (intercept) effects were included to account for non‐independence of repeat measures of skin colour collected from the same fish.

All models were fitted using restricted maximum likelihood (REML) with the significance of predictor terms determined by either Wald F testing (GLS models) or likelihood ratio testing (LME and GAMM models). Where residual plots indicated a lack of residual independence, we incorporated the dependency into the model using either “VarIdent” (the GLS and LME models) or “VarPower” (the GAMM models) variance structures (Zuur *et al*., 2009). GLS and LME models were fitted using the “nlme” R package. GAMM models were fitted using the “mgcv” package. Final model coefficient values are presented in Appendix S1.

## 3 RESULTS

In visual terms, non‐crowded, unstressed *S. scombrus* skin was predominately green (Figure 1). The mean (± 95% c.i.) CIE colour space values for control, uncrowded fish were: L* = 47.67 ± 1.06; a* = −9.27 ± 1.16; and b* = 17.17 ± 1.07.

Once exposed to crowding, there was evidence for stress‐induced alteration to skin colour in the L* and a* CIE colour space components (significance of the “trial” and “monitoring period” predictor variables: < 0.01 in all cases). Fish exposed to crowding, as well as moribund individuals, tended to have marginally lower lightness (L*, Supporting Information Figure S1) and display a shift in colour from red towards greenness (a*, Supporting Information Figure S3) during and/or following crowding. The size of these effects was relatively small.

However, notable alteration to skin colour occurred in the b* yellow‐blue axis (Supporting Information Figure S2). Crowded *S. scombrus* skin tended to increase in blueness (Figure 1) during exposure to the stressor (Supporting Information Figure S2). This increase was especially pronounced for moribund individuals and during the “High & Prolonged 1” trial (Supporting Information Figure S2), and is supported by the highly significant differences in CIE b* values between trials (*F* = 38.17, *df* = 6, *P* < 0.0001) and between monitoring periods (*F* = 6.21, *df* = 4, *P* < 0.0001). Further increases in blueness were evident at 2 h post‐crowding, with some evidence of recovery by *c*. 24 h (Supporting Information Figure S2). There were also indications of significant differences in blueness for uncrowded fish between control trials, but again, the effect size was small and negligible in comparison to the crowding‐induced colour change.

**FIGURE 2:**
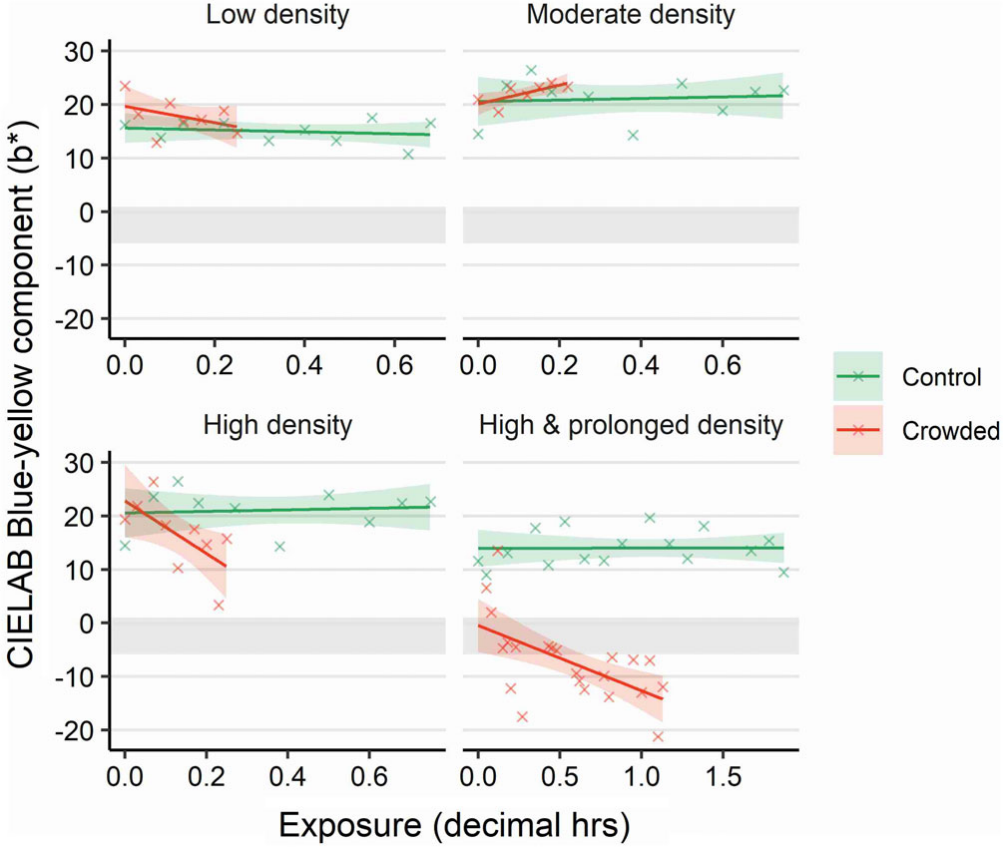
Atlantic mackerel (*Scomber scombrus*) skin colour change (in terms of blue‐yellowness in CIELAB colour space; blue −ve and yellow +ve) during exposure to crowding stress. Groups of wild‐caught *S. scombrus* were crowded at different densities and durations in sea cages. Control cages were not crowded. Individuals were photographed in air throughout stressor exposure under standard lighting conditions. Photographs were digitally analysed for colour. The coloured shaded areas indicate model derived 95% c.i., with the underlying data indicated as crosses. For comparison, the b* 95% c.i. for moribund individuals is included (grey shaded area). Note the different *x*‐axis scale for the high & prolonged density group

The degree of colour change towards blueness during crowding events depended on exposure duration and the applied crowding density (significance of “trial × exposure” interactive term: *F* = 3.78, *df* = 6, *P* = 0.003). Longer crowding exposure times resulted in increased blueness values for trials in which crowding was applied, whereas skin colour in control trials remained relatively stable (Figure 2). There was a general tendency for higher densities to result in greater rates of change towards blueness (Figure 2). For the “Low,” “High” and “High & Prolonged” trials, the model predicted the rate of skin colour change as 0.25, 0.82 and 0.20 reduction in b* units per min, respectively. The “moderate” trial was an exception to the trend, for which the model predicted reduced levels of blueness with increasing exposure time (Figure 2, 0.30 increase in b* units per min). Nonetheless, the range of this apparent effect is small (18.5 to 24.0) and bounded by the 95% c.i. for the respective control values.

**FIGURE 3:**
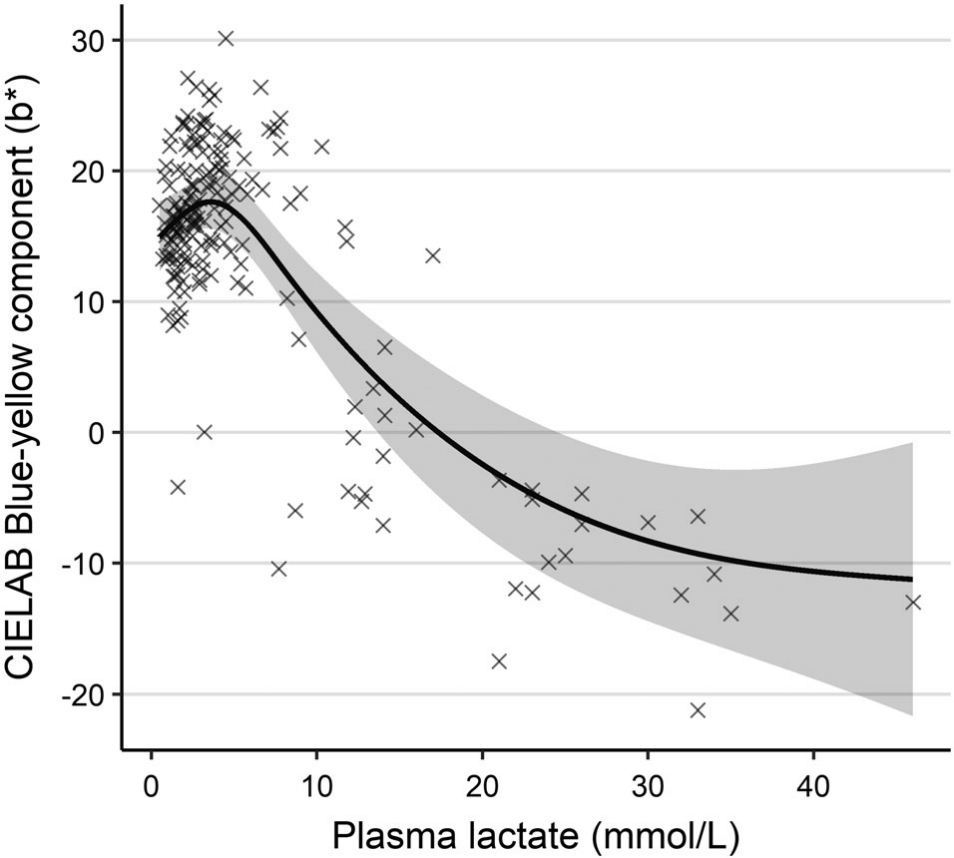
The relationship between blood plasma lactate concentration and skin colour (in terms of blue‐yellowness in CIELAB colour space; blue −ve and yellow +ve) in Atlantic mackerel (*Scomber scombrus*) during crowding stress trials. Groups of wild‐caught *S. scombrus* were crowded at different densities and durations in sea cages and were sampled prior to, during and after stressor exposure. Individuals were photographed in air and had blood collected immediately after. Photographs were digitally analysed for colour. The shaded area indicates the model derived 95% c.i.. Crosses indicate the underlying data

CIE b* values in individual fish were significantly correlated (LRT = 30.30, *df* = 5, *P* < 0.001) to plasma lactate levels. Higher lactate levels were associated with reductions in b* values (Figure 3), that is, a shift in colour towards blueness. Conversely, plasma glucose and cortisol were not useful predictors of an individual's skin colour (*P* > 0.05 in both cases).

**FIGURE 4:**
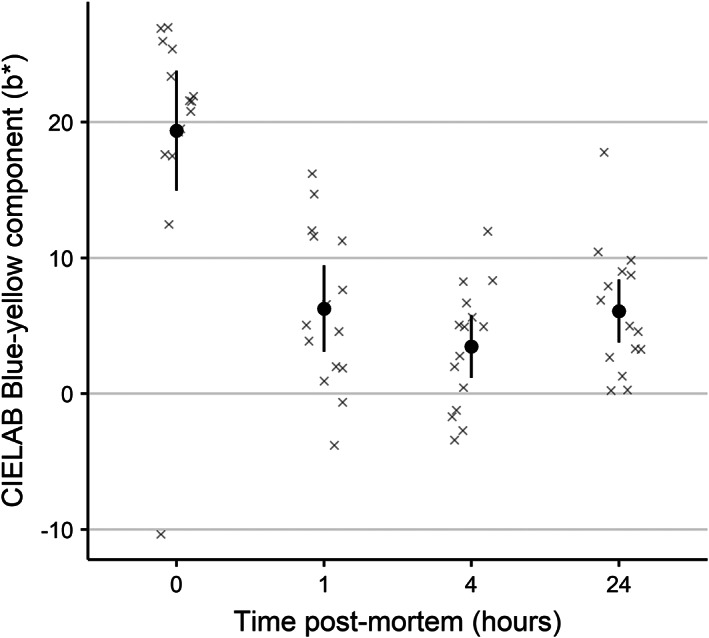
Stability of Atlantic mackerel (*Scomber scombrus*) skin colour (in terms of blue‐yellowness in CIELAB colour space; blue −ve and yellow +ve) after death. Wild captured *S. scombrus* were caught from sea cages, and immediately brain spiked prior to storage in ambient sea water. The same individuals were photographed over time in air. Photographs were digitally analysed for colour. Points and whiskers indicate model derived mean and 95% c.i., with the underlying data indicated as crosses

*Post‐mortem S. scombrus* skin colour (CIE b* values) was not influenced by light storage conditions (LRT = 0.13, *df* = 1, *P* = 0.72). Despite this, fish significantly increased in blueness (LRT = 24.32, *df* = 3, *P* < 0.001) within 1 h of death and remained at comparable levels until at least 24 h after death (Figure 4).

## 4 DISCUSSION

The presented data support previous qualitative observations that *S. scombrus* skin colour can change from predominately green to predominately blue in response to stressors (Holeton *et al*., 1982; Lockwood *et al*., 1983; Pawson & Lockwood, 1980; Swift, 1982, 1983). However, our benign husbandry procedures (as evidenced by several physiological metrics and the complete lack of mortality in control trials, Anders *et al*., 2021) allow us to extend the findings of previous authors and conclude that crowding stress can induce quantifiable changes in *S. scombrus* skin colour. The results also support a conclusion that the degree of colour change reflects the amount of stress received, as evidenced by the correlations to physiological stress and to the intensity and duration of crowding, and that the change occurs within the typical time frame of crowding events in the fishery. The findings that *S. scombrus* rapidly turn blue after death, and that non‐moribund fish may begin to recover from any colour change within 24 h post‐crowding, imply that colour change as a stress indicator is reliable only for live fish during exposure to the crowding stressor.

We did not establish the causal mechanism behind the observed skin colour change. In fish, colour change occurs *via* neural and hormonally mediated alterations to chromatophores. The rapidity of the colour change would suggest that physiologically mediated changes to pigments or light‐reflecting crystals within chromatophores were responsible, rather than slower morphological changes (as reviewed by Sköld *et al*., 2016). The strong correlation between blueness and plasma lactate may indicate that the colour change is related to, or at least marked by, a state of functional hypoxia within the animal's skin tissue. Such hypoxia likely arose due to reductions in ambient oxygen levels (probably due to restricted water exchange through the crowded biomass of fish) and crowding‐induced increases in behavioural activity beyond aerobic thresholds (Anders *et al*., 2021; Anders, Howarth, *et al*., 2019). The finding that unstressed (and therefore presumably not oxygen deficient) fish rapidly turned blue once dead (after which ventilation ceases and oxygen levels can be expected to decrease) also supports such a hypoxia‐related explanation. In carp (*Cyprinus carpio*) and common sole (*Solea solea)*, asphyxia resulted in changes to body colouration (Nagai & Iriki, 1978; Wilhelm, 1969). These observations may also be explained by hypercapnia, with an increase in the partial pressure of carbon dioxide (*p*CO_2_) and an associated reduction in pH, in both the ambient sea water and animals' tissues, occurring contemporaneously with hypoxia. Alternatively, a variety of neuroendocrine responses to stress are known to induce colour change in fish (reviewed in Nilsson Sköld *et al*., 2013). For instance, increases in plasma levels of α‐melanophore‐stimulating hormone (αMSH) result from stress‐induced activation of the melanocortin system (Cal *et al*., 2017; Cerdá‐Reverter *et al*., 2011). αMSH has been associated with skin colour change in many fish species (Nilsson Sköld *et al*., 2013). Rapid colour change can also result from changes to chromatophores *via* sympathetic neural mechanisms (Burton, 2008). We suggest that similar experiments to those described by Oshima *et al*. (1989) (including monitoring of *p*O_2_, *p*CO_2_ and pH) be performed on *S. scombrus* to better understand the physiological mechanism that induces the observed colour change.

Due to the challenges in working in a large, at‐sea mesocosm experimental set‐up, we were unable to replicate the applied densities. We are therefore unable to characterise any variability in the skin colour response that may occur between different schools of *S. scombrus*. Nonetheless, the correlations between increasing crowding exposure/intensity and blueness, as well as with physiological disturbance in individual fish, indicate that the conclusions of this study are still valid. Nevertheless, it would be useful for future work to determine how repeatable the skin colour change response is and what factors modify the response. Observations of skin colour from real purse seine catches are also required to confirm that the response occurs in real‐world situations and in wild captured fish. If the skin colour change is physiologically mediated, potential intraspecific differences in the colour change response between different stocks of *S. scombrus* should also be investigated to determine the applicability of the stress indicator for fisheries that operate in different areas.

Although we monitored post‐crowding survival rates in trials in which the colour change was observed, we did so at the population level rather than at the level of individuals (apart from the moribund individuals). It is therefore difficult to directly link the degree of skin colour change to specific welfare outcomes of relevance to the wild capture fishing industry, such as mortality and negative flesh quality implications. Such investigations should be the subject of future work to further increase the usefulness of skin change as a stress indicator, but would be difficult to perform using the experimental set‐up described in the current study. Smaller, tank‐based experiments may therefore be required in which the outcomes of stress for individual fish can be more practically monitored.

To be useful, any potential stress indicator should be quantifiable, respond rapidly and correlate to the amount of stress received (Noble *et al*., 2018). The results of this study indicate that skin colour change in response to crowding stress corresponds well to these criteria in that: (a) it can be objectively quantified *via* digital methods; (b) it occurs during stressor exposure (and within the typical time frame of crowding exposure in the purse seine fishery); and (c) the degree of colour change reflects the amount of stress received, as well underlying physiological disturbances. A useful indicator for use in wild capture fishing must also be practical to measure during dynamic fishing operations. The skin colour change is easily visible (as opposed to disturbances to blood physiology which require time‐consuming and invasive sampling, or behavioural indicators that can be difficult to interpret, Sopinka *et al*., 2016), so it also conforms with the criteria of being practical. The technological challenge for the future will be to devise solutions (such as new observation platforms and automated image analysis) that can rapidly and reliably monitor skin colour in captured *S. scombrus* schools inside purse seines. If this can be achieved, fishers could make informed real‐time decisions during fishing operations that would mitigate capture stress and improve fish welfare. This may lead to improvements to the quality of landed catches through improved fillet quality, as well as to sustainability of the fishery through increased survivability of released catches.

## AUTHOR CONTRIBUTIONS

G.M.T., M.S.B. and J.R.M. conceived the study. M.B. and N.A. carried out the crowding experiments and acquired the images used in the study. Data analysis was conducted by M.S.B (image analysis), N.A. (statistical modelling) and M.B. (data management). G.M.T and M.B. coordinated the study. M.B. sourced the funding.. All authors contributed to the experimental design and writing of the manuscript.

## Supporting information


**APPENDIX S1.** Supporting information.
