# Supplementary material for: Atlantic mackerel ( Scomber scombrus ) change skin colour in response to crowding stress
Source: J Fish Biol. 2022 Jan 23;100(3):738–47. doi: 10.1111/jfb.14987 (PMC9306841; doi:10.1111/jfb.14987)
Supplement: Supplementary file 1 — APPENDIX S1. Supporting information. [file JFB-100-738-s001.docx]

**Supporting Information: Model coefficient values.**

**The effect of crowding on skin colour lightness (*L**) in Atlantic mackerel**

Model form (simplified R syntax):

gls(L ~ Monitoring period + Trial)

Coefficient table (adjustments relative to coefficient estimate for “Control 1”):

| **Term** | **Coefficient** | **Std. error** | **t-value** | **p-value** |
| --- | --- | --- | --- | --- |
| (Intercept) | 46.618 | 1.41 | 33.061 | 0 |
| Monitoring period (2hrs post-treatment) | -0.194 | 1.256 | -0.155 | 0.877 |
| Monitoring period (Pre-treatment) | 2.624 | 1.204 | 2.179 | 0.031 |
| Monitoring period (Termination) | -1.207 | 1.475 | -0.819 | 0.414 |
| Monitoring period (Treatment) | 2.153 | 1.088 | 1.979 | 0.049 |
| Trial (Control 2) | 0.688 | 1.346 | 0.511 | 0.61 |
| Trial (Control 3) | -1.664 | 1.318 | -1.263 | 0.208 |
| Trial (High & Prolonged) | -2.475 | 1.361 | -1.818 | 0.071 |
| Trial (Low) | 0.6 | 1.38 | 0.435 | 0.664 |
| Trial (Moderate) | -3.32 | 1.366 | -2.431 | 0.016 |
| Trial (High) | 0.703 | 1.326 | 0.53 | 0.597 |

Visualization


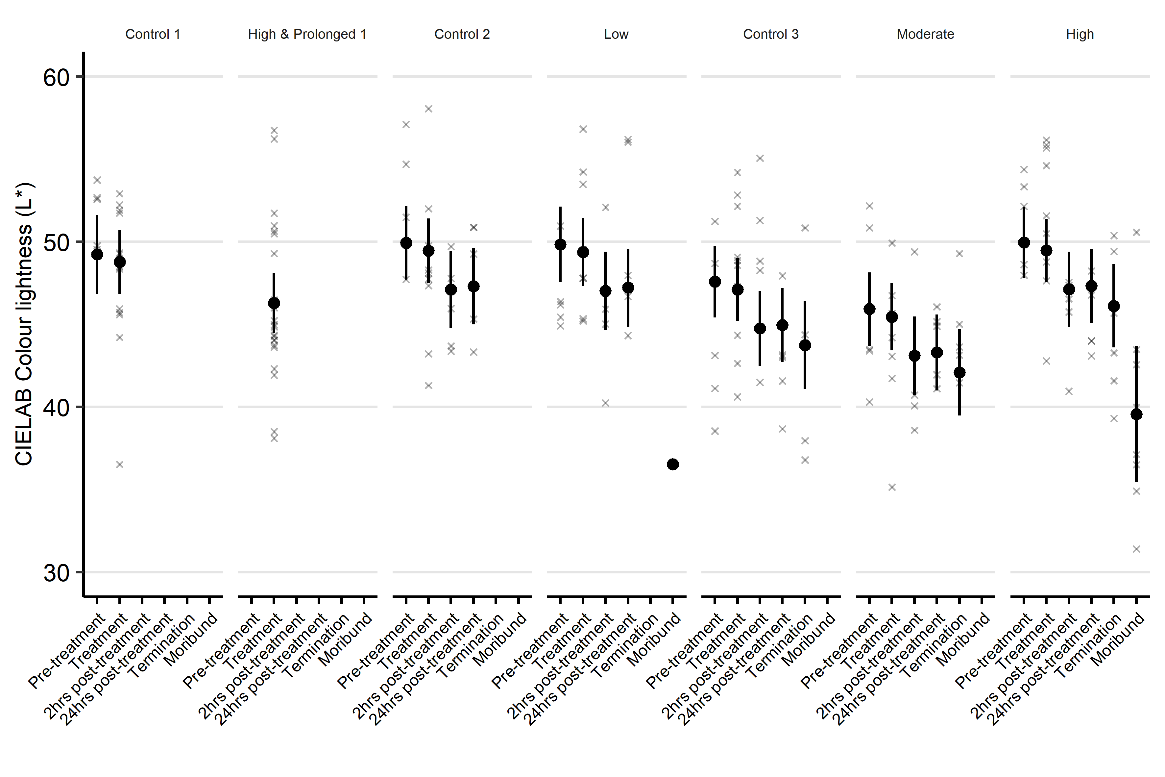
**Supplementary Figure S1:** The influence of crowding stress on skin colour lightness (CIELAB colour space; blue -ve and yellow +ve) of Atlantic mackerel (*Scomber scombrus*). Groups of wild caught mackerel were crowded at different densities and durations in sea cages. Control cages were not crowded. Individuals were photographed in air under standard lighting conditions prior to, during and after stressor exposure. Photographs were digitally analysed for colour. Points and whiskers indicate model derived mean and 95% confidence intervals, with the underlying dataset indicated as crosses.

**The effect of crowding on the blue-yellow colour component (*b**) of Atlantic mackerel skin**

Model form (simplified R syntax):

gls(b ~ Monitoring period + Trial,

weights = varIdent(form = ~ 1 | Trial))

Coefficient table (adjustments relative to coefficient estimate for “Control 1”):

| **Term** | **Coefficient** | **Std. error** | **t-value** | **p-value** |
| --- | --- | --- | --- | --- |
| (Intercept) | 14.603 | 1.307 | 11.169 | 0 |
| Monitoring period (2hrs post-treatment) | -2.087 | 1.272 | -1.64 | 0.103 |
| Monitoring period (Pre-treatment) | 0.638 | 1.202 | 0.531 | 0.596 |
| Monitoring period (Termination) | -1.479 | 1.564 | -0.945 | 0.346 |
| Monitoring period (Treatment) | -0.14 | 1.093 | -0.128 | 0.898 |
| Trial (Control 2) | 1.569 | 1.113 | 1.409 | 0.161 |
| Trial (Control 3) | 6.06 | 1.274 | 4.755 | 0 |
| Trial (High & Prolonged) | -21.622 | 1.825 | -11.848 | 0 |
| Trial (Low) | 0.954 | 1.374 | 0.694 | 0.488 |
| Trial (Moderate) | 5.588 | 1.214 | 4.605 | 0 |
| Trial (High) | 2.914 | 1.351 | 2.157 | 0.032 |

Visualization


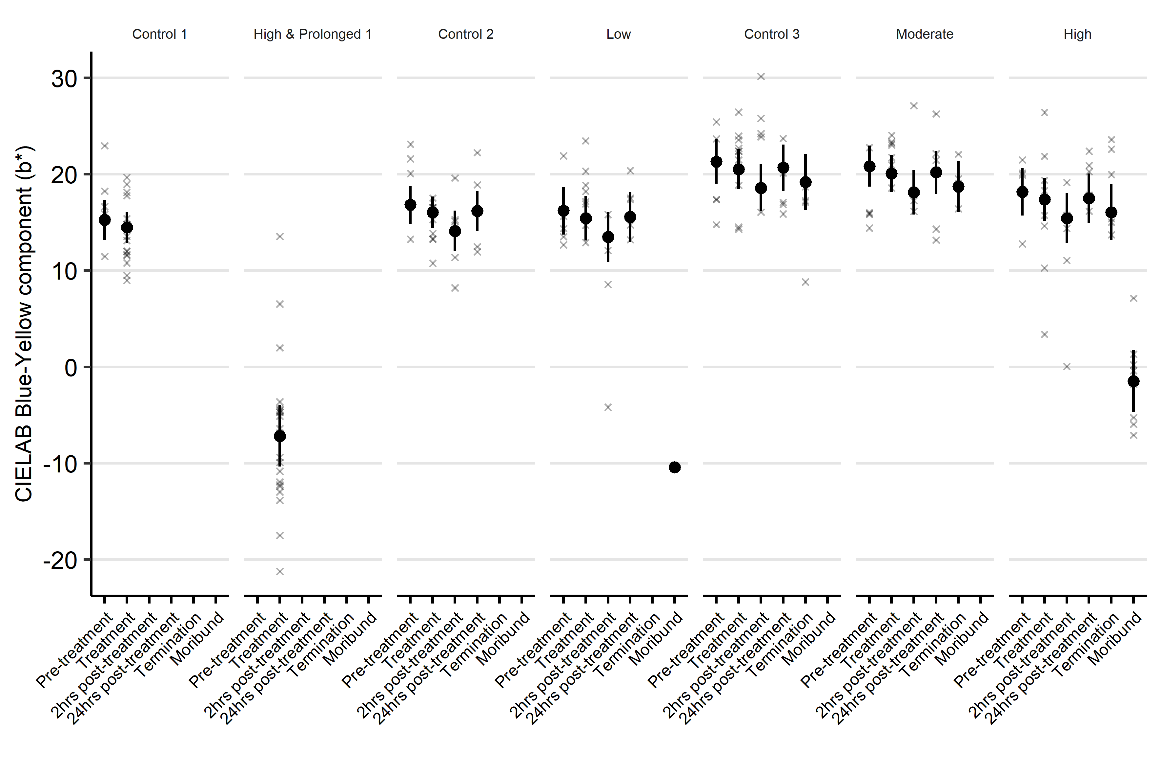


**Supplementary Figure S2:** The influence of crowding stress on the colour of Atlantic mackerel (*Scomber scombrus*) skin, in terms of blue–yellowness (CIELAB colour space; blue -ve and yellow +ve). Groups of wild caught mackerel were crowded at different densities and durations in sea cages. Control cages were not crowded. Individuals were photographed in air under standard lighting conditions prior to, during and after stressor exposure. Photographs were digitally analysed for colour. Points and whiskers indicate model derived mean and 95% confidence intervals, with the underlying dataset indicated as crosses.

**The effect of crowding on the red-green colour component (*a**) of Atlantic mackerel skin**

Model form (simplified R syntax):

gls(a ~ Vitality + Monitoring period + Trial,

weights = varIdent(form = ~ 1 | Trial))

Coefficient table (adjustments relative to coefficient estimate for “Control 1”):

| **Term** | **Coefficient** | **Std. error** | **t-value** | **p-value** |
| --- | --- | --- | --- | --- |
| (Intercept) | -12.102 | 0.93 | -13.009 | 0 |
| Vitality (measured) | -1.171 | 0.494 | -2.372 | 0.019 |
| Monitoring period (2hrs post-treatment) | -1.664 | 0.889 | -1.871 | 0.063 |
| Monitoring period (Pre-treatment) | 0.689 | 0.843 | 0.818 | 0.414 |
| Monitoring period (Termination) | 1.565 | 1.225 | 1.278 | 0.203 |
| Monitoring period (Treatment) | -0.277 | 0.766 | -0.361 | 0.718 |
| Trial (Control 2) | 1.47 | 0.761 | 1.933 | 0.055 |
| Trial (Control 3) | 8.022 | 1.158 | 6.93 | 0 |
| Trial (High & Prolonged) | 0.856 | 0.937 | 0.913 | 0.362 |
| Trial (Low) | 3.432 | 0.786 | 4.367 | 0 |
| Trial (Moderate) | 7.277 | 0.893 | 8.148 | 0 |
| Trial (High) | 3.553 | 0.927 | 3.834 | 0 |

Visualization


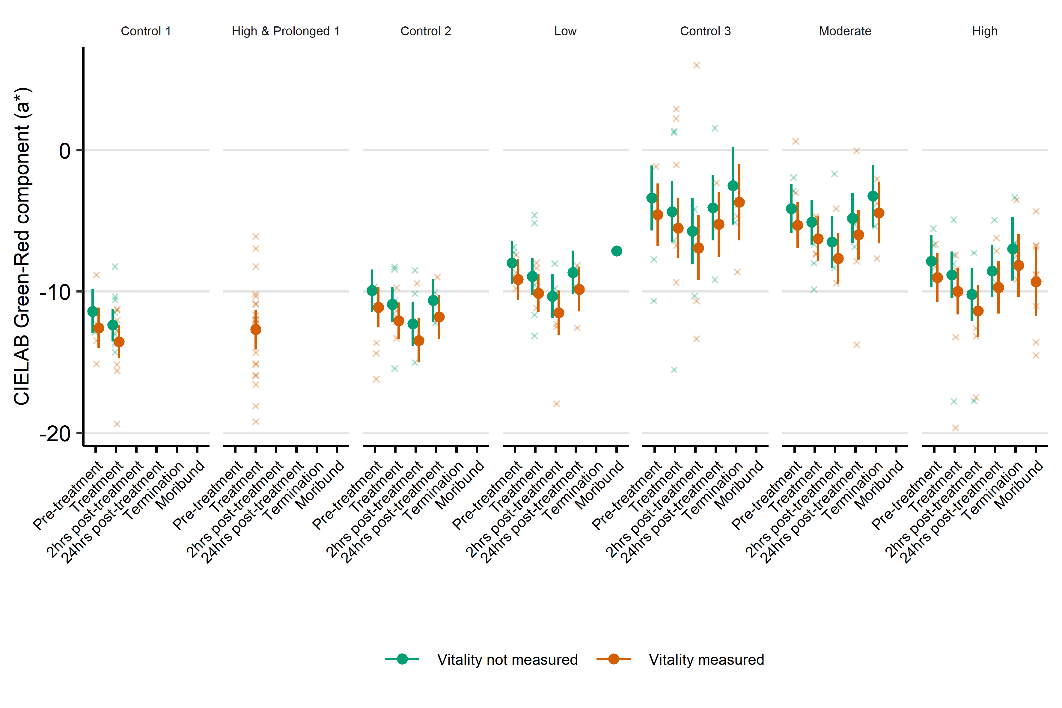


**Supplementary Figure S3:** The influence of crowding stress on the colour of Atlantic mackerel (*Scomber scombrus*) skin, in terms of green–redness (CIELAB colour space; blue -ve and yellow +ve). Groups of wild caught mackerel were crowded at different densities and durations in sea cages. Control cages were not crowded. Individuals were photographed in air under standard lighting conditions prior to, during and after stressor exposure. Photographs were digitally analysed for colour. Points and whiskers indicate model derived mean and 95% confidence intervals, with the underlying dataset indicated as crosses.

**Atlantic mackerel skin colour change during exposure to crowding - blue-yellow component (*b**)**

Model form (simplified R syntax):

gls(b ~ Trial + Exposure + Trial × Exposure,

weights = varComb(varExp(form = ~ Exposure), varIdent(form = ~ 1 | Trial)))

Coefficient table (adjustments relative to coefficient estimate for “Control 1”):

| **Term** | **Coefficient** | **Std. error** | **t-value** | **p-value** |
| --- | --- | --- | --- | --- |
| (Intercept) | 13.933 | 1.757 | 7.93 | 0 |
| Trial (Control 2) | 1.661 | 2.254 | 0.737 | 0.464 |
| Trial (Control 3) | 6.606 | 2.937 | 2.249 | 0.028 |
| Trial (High & Prolonged) | -14.383 | 3.09 | -4.655 | 0 |
| Trial (Low) | 5.733 | 2.67 | 2.148 | 0.035 |
| Trial (Moderate) | 6.169 | 2.039 | 3.025 | 0.004 |
| Trial (High) | 8.888 | 3.885 | 2.288 | 0.025 |
| Exposure | 0.046 | 1.447 | 0.032 | 0.975 |
| Trial (Control 2) × Exposure | -1.835 | 3.547 | -0.517 | 0.607 |
| Trial (Control 3) × Exposure | 1.404 | 5.26 | 0.267 | 0.79 |
| Trial (High & Prolonged) × Exposure | -12.249 | 3.853 | -3.179 | 0.002 |
| Trial (Low) × Exposure | -15.392 | 13.484 | -1.141 | 0.258 |
| Trial (Moderate) × Exposure | 17.639 | 7.713 | 2.287 | 0.025 |
| Trial (High) × Exposure | -49.186 | 21.945 | -2.241 | 0.028 |

Model presented graphically as Figure 2 in the main article.

**Correlation between the blue-yellow colour component (*b**) of Atlantic mackerel skin and plasma lactate**

Model form (simplified R syntax):

gamm(b ~ s(Lactate, bs="cr", k=4),

random = list(Trial = ~ 1, Monitoring period^§^ = ~1),

weights = varPower(form = ~ Lactate))

^§^ Monitoring period encoded uniquely across different trials

GAM component coefficient table

| **Term** | **Coefficient** | **Std. error** | **t-value** | **p-value** |
| --- | --- | --- | --- | --- |
| (Intercept) | 13.402 | 0.951 | 14.09 | <0.001 |

Approximate significance of smooth terms

| **Term** | **edf** | **Reference df** | **F-value** | **p-value** |
| --- | --- | --- | --- | --- |
| s(Lactate) | 2.891 | 2.891 | 19.95 | <0.001 |

LME component coefficient table

| **Term** | **Coefficient** | **Std. error** | **t-value** | **p-value** |
| --- | --- | --- | --- | --- |
| (Intercept) | 13.402 | 0.954 | 14.053 | <0.001 |
| Lactate | -24.849 | 5.142 | -4.833 | <0.001 |

Model presented graphically as Figure 3 in the main article.

**Correlation between the blue-yellow colour component (*b**) of Atlantic mackerel skin and plasma cortisol**

Model form (simplified R syntax):

gamm(b ~ s(Cortisol, bs="cr", k=-1),

random = list(Trial = ~ 1, Monitoring period^§^ = ~1))

^§^ Monitoring period encoded uniquely across different trials

GAM component coefficient table

| **Term** | **Coefficient** | **Std. error** | **t-value** | **p-value** |
| --- | --- | --- | --- | --- |
| (Intercept) | 14.018 | 2.283 | 6.14 | <0.001 |

Approximate significance of smooth terms

| **Term** | **edf** | **Reference df** | **F** | **p-value** |
| --- | --- | --- | --- | --- |
| s(Cortisol) | 2.481e-05 | 9 | <0.001 | 0.981 |

LME component coefficient table

| **Term** | **Coefficient** | **Std. error** | **t-value** | **p-value** |
| --- | --- | --- | --- | --- |
| (Intercept) | 14.018 | 2.283 | 6.140 | <0.001 |

Visualization


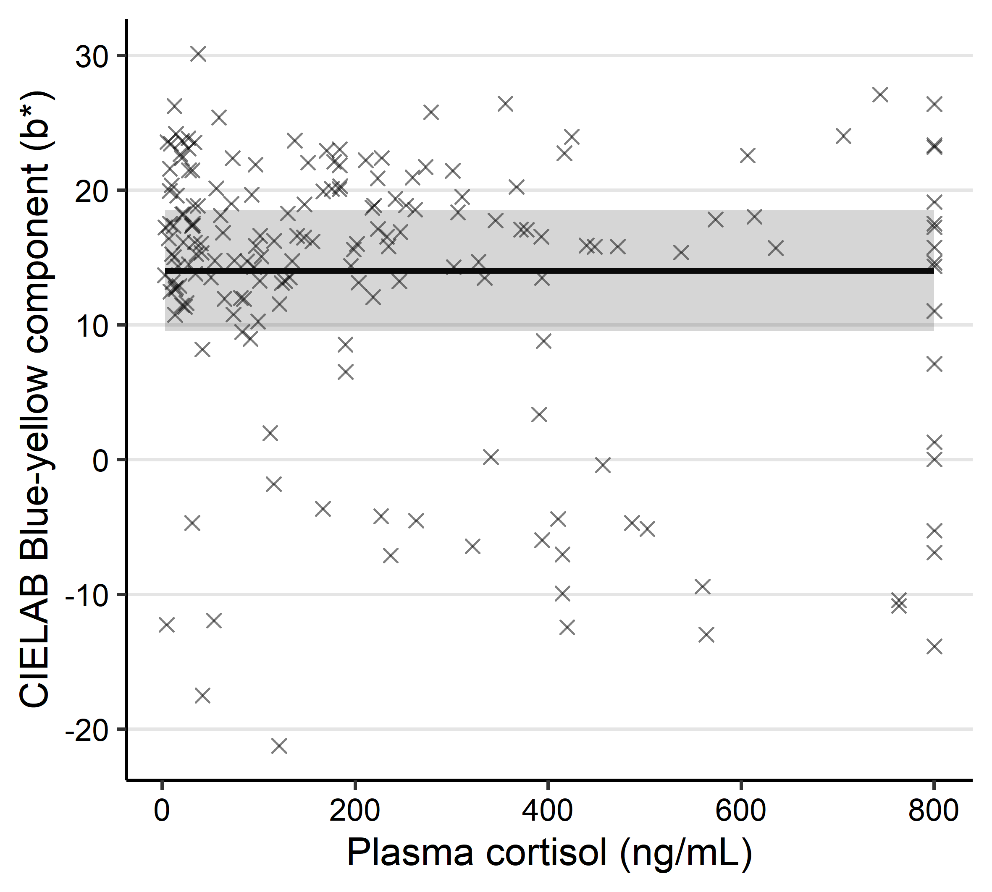


**Correlation between the blue-yellow colour component (*b**) of Atlantic mackerel skin and plasma glucose**

Model form (simplified R syntax):

gamm(b ~ s(Glucose, bs="cr", k=3),

random = list(Trial = ~ 1, Monitoring period^§^ = ~1),

weights=varPower(form = ~ Glucose))

^§^ Monitoring period encoded uniquely across different trials

GAM component coefficient table

| **Term** | **Coefficient** | **Std. error** | **t-value** | **p-value** |
| --- | --- | --- | --- | --- |
| (Intercept) | 13.670 | 2.662 | 5.134 | <0.001 |

Approximate significance of smooth terms

| **Term** | **edf** | **Reference df** | **F** | **p-value** |
| --- | --- | --- | --- | --- |
| s(Cortisol) | 1.726 | 1.726 | 0.99 | 0.307 |

LME component coefficient table

| **Term** | **Coefficient** | **Std. error** | **t-value** | **p-value** |
| --- | --- | --- | --- | --- |
| (Intercept) | 13.669 | 2.669 | 5.120 | <0.001 |
| Glucose | 0.192 | 2.356 | 0.081 | 0.935 |

Visualization


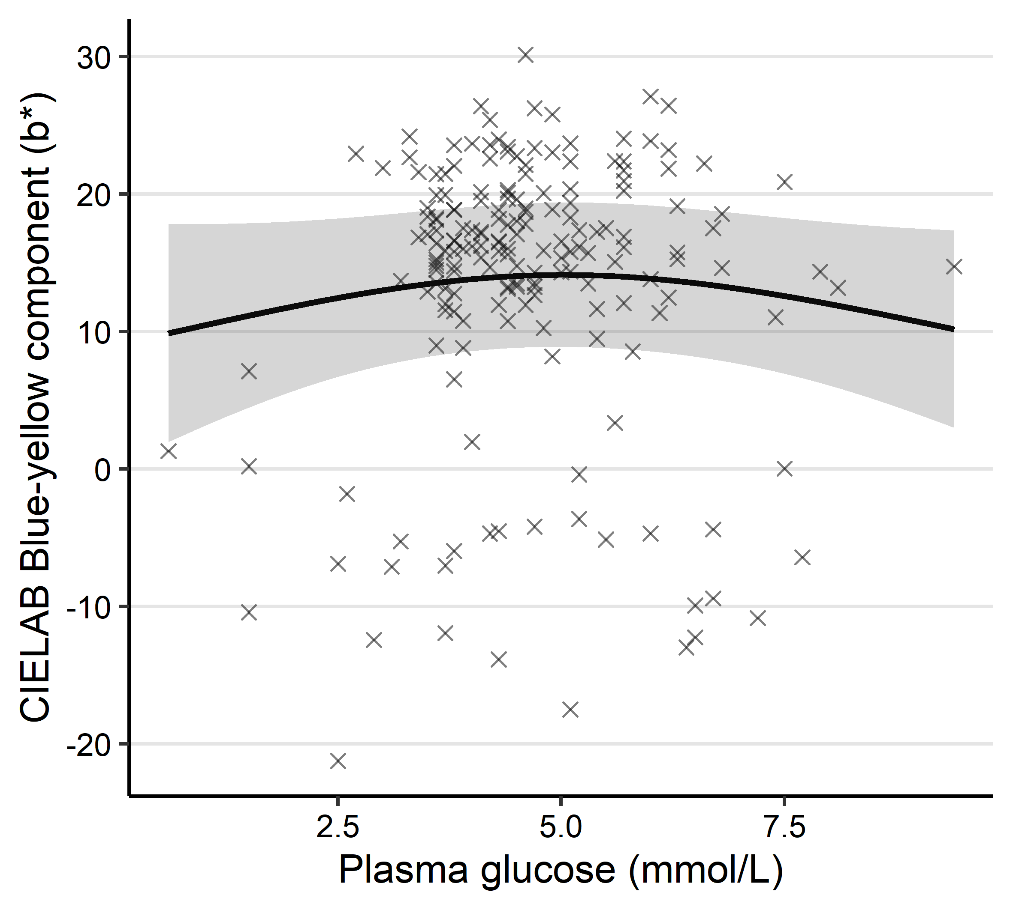


**Stability of the blue-yellow colour component (*b**) of Atlantic mackerel skin *post-mortem***

Model form (simplified R syntax):

lme(b ~ Time.post.mortem,

random = ~ 1 | Fish.ID,

weights = varIdent(form = ~ 1 | Time.post.mortem))

Coefficient table (adjustments relative to coefficient estimate for “Time post mortem [0 hour]”):

| **Term** | **Coefficient** | **Std. error** | **df** | **t-value** | **p-value** |
| --- | --- | --- | --- | --- | --- |
| (Intercept) | 19.363 | 2.251 | 42 | 8.604 | <0.001 |
| Time.post.mortem (1 hour) | -13.104 | 2.609 | 42 | -5.023 | <0.001 |
| Time.post.mortem (4 hours) | -15.898 | 2.357 | 42 | -6.745 | <0.001 |
| Time.post.mortem (24 hours) | -13.283 | 2.362 | 42 | -5.624 | <0.001 |

Model presented graphically as Figure 4 in the main article.
